# Total resection of a giant retroperitoneal and mediastinal ganglioneuroma—case report and systematic review of the literature

**DOI:** 10.1186/s12957-020-02016-1

**Published:** 2020-09-18

**Authors:** Patrick Kirchweger, Helwig Valentin Wundsam, Ines Fischer, Christiane Sophie Rösch, Gernot Böhm, Oleksiy Tsybrovskyy, Vedat Alibegovic, Reinhold Függer

**Affiliations:** 1 Department of General and Visceral Surgery, Ordensklinikum Linz – Barmherzige Schwestern, Linz, Austria; 2 Medical Faculty, Johannes Kepler University Linz, Linz, Austria; 3 Department of General, Visceral, Vascular, Thoracic and Transplantation Surgery, Ordensklinikum Linz – Elisabethinen, Linz, Austria; 4 Department of Radiology, Ordensklinikum Linz – Barmherzige Schwestern, Linz, Austria; 5 Department of Pathology – Vinzenz Pathologieverbund, Ordensklinikum Linz, Linz, Austria

**Keywords:** Case report, Largest, Thoracoabdominal, Ganglioneuroma, Systematic review

## Abstract

**Background:**

Ganglioneuromas (GNs) are extremely rare, slowly growing, benign tumors that can arise from Schwann cells, ganglion cells, and neuronal or fibrous tissues. Due to their origin from the sympathetic neural crest, they show neuroendocrine potential; however, most are reported to be hormonally inactive. Nevertheless, complete surgical removal is recommended for symptom control or for the prevention of potential malignant degeneration.

**Case Report:**

A 30-year-old female was referred to our oncologic center due to a giant retroperitoneal and mediastinal mass detected in computed tomography (CT) scans. The initial symptoms were transient nausea, diarrhea, and crampy abdominal pain. There was a positive family history including 5 first- and second-degree relatives. Presurgical biopsy revealed a benign ganglioneuroma. Total resection (TR) of a 35 × 25 × 25 cm, 2550-g tumor was obtained successfully via laparotomy combined with thoracotomy and partial incision of the diaphragm. Histopathological analysis confirmed the diagnosis. Surgically challenging aspects were the bilateral tumor invasion from the retroperitoneum into the mediastinum through the aortic hiatus with the need of a bilateral 2-cavity procedure, as well as the tumor-related displacement of the abdominal aorta, the mesenteric vessels, and the inferior vena cava. Due to their anatomic course through the tumor mass, the lumbar aortic vessels needed to be partially resected. Postoperative functioning was excellent without any sign of neurologic deficit.

**Conclusion:**

Here, we present the largest case of a TR of a GN with retroperitoneal and mediastinal expansion. On review of the literature, this is the largest reported GN resected and was performed safely. Additionally, we present the first systematic literature review for large GN (> 10 cm) as well as for resected tumors growing from the abdominal cavity into the thoracic cavity.

## Background

Ganglioneuromas (GNs) are extremely rare (1/1,000,000), slowly growing, benign tumors that can arise from Schwann cells, ganglion cells, and neuronal (0.1–0.5% of neurogenic tumors) or fibrous tissues [1–3]. First described by Loretz in 1870, they are most commonly seen in pediatric populations, with 60% of total diagnoses occurring prior to the age of 20. The median age at the time of the diagnosis is reported to be approximately 7 years [4]. Ganglioneuromas, in general, occur more frequently in females than in males with a ratio of about 3:2 [5]. As these tumors are generally diagnosed due to compressive symptoms and consequently resected in children, there is usually a natural limit to tumor size given by the available space within the body cavity. The biggest resected GN assessable through literature research up to now showed a maximum diameter of approximately 23 cm [6] in a 42-year-old patient located solely thoracically [6]. Located most commonly in the posterior mediastinum (41.5%) or retroperitoneally (37.5%), ganglioneuromas can be found in the adrenal glands (21%), in the neck (8%), retropharyngeally, or more rarely in the sella turcica [7–10]. Computed tomography (CT) or magnetic resonance imaging (MRI) represents the gold standard for diagnosis and estimation of tumor extent. Microscopically, the absence of mitotic figures, intermediate cells, neuroblasts, or necrosis distinguishes a GN from their main differential diagnoses, ganglioneuroblastoma and paraganglioma, which are considered functioning tumors [11, 12]. Ganglioneuromas show neuroendocrine potential which is attributable to their origin from the sympathetic neural crest, but the majority of them are reported to be hormonally inactive [13]. However, GNs have shown to have a secretory function in up to 39% of cases in some studies [2]. Elevated levels of metanephrine, catecholamine, vasoactive intestinal peptide (VIP), dopamine, cortisol, homovanillic acid (HVA), or vanillylmandelic acid (VMA) can be potentially detected in the blood or urine of patients, especially if the adrenal gland is involved. Currently, metanephrine detection in the plasma of patients is regarded as the gold standard for the detection of catecholamine-releasing tumors. These tumors are much more likely to cause symptoms such as hypertensive crisis, diarrhea, virilization due to hormonal imbalance, and depressive disorders [13–17]. Some studies indicate an association between a diagnosed ganglioneuroma and genetic diseases like multiple endocrine neoplasia type 2 or neurofibromatosis type 1 or 2 [3]. Malignant degeneration of a ganglioneuroma occurs even more rarely, with the highest prevalence occurring when tumors are penetrating into the spinal canal via the neural foramen, with transformation into neuroblastoma [18–21]. Thus, complete surgical removal is recommended for symptom control or prevention of potential malignant degeneration [22]. Furthermore, subsequent long-term follow-up including imaging controls is mandatory to prevent potential relapse, especially when only partial tumor removal was achieved. There is no need for neoadjuvant or adjuvant antineoplastic treatment [1]. Additionally, prognosis after total tumor resection is deemed to be excellent, although surgical morbidity has to be taken into account especially when dealing with large GN [2].

We report the largest resected ganglioneuroma expanding from the retroperitoneum to the thoracic cavity. Currently, there is no systematic review of large resected ganglioneuroma or thoracoretroperitoneal tumors. We conducted a literature review adhering to the PRISMA guidelines to address this issue and to place our findings in the context of the current literature.

## Methods

First, we present our patient in terms of preoperative imaging, surgical therapy, and postoperative outcome. The case report was conducted adhering to the CARE guidelines [23]. The required checklist is provided in the supplementary material (Supplementary file 1).

Second, in January 2020, a systematic literature research for big GN (> 10 cm) was conducted using the terms <large> OR <largest> OR <big> OR <biggest> OR <giant> OR <huge> OR <massive> OR <retroperitoneal> OR <mediastinal> AND <resected> OR <resection> OR <surgery> AND <ganglioneuroma> on PubMed and MEDLINE research to isolate all cases of the largest resected GN in all locations as well as GN with retroperitoneal and mediastinal expansion.

Third, a systematic literature research was conducted for the identification of all resected tumors with expansion from the retroperitoneum to the mediastinum by using the MeSH terms <tumor>, <mediastinum>, <retroperitoneum>, and <resection> as well as <thoracoabdominal>, <tumor>, and <resection> on PubMed and MEDLINE in February 2020. The papers were reviewed, and reports of gastric or esophageal cancers as well as spinal cord surgery were excluded.

No further specific eligibility criteria were used to address all reported cases and possibly existing case series, except the ones mentioned above and visualized in Fig. 4.

Systematic reviews were conducted in accordance with the search adhering to the PICOS strategy, and reporting was done subject to the regulations of the Preferred Reporting Items for Systematic Reviews and Meta-Analyses (PRISMA) guidelines [24]. The complete checklist is provided in the supplementary material (Supplementary file 2).

### Case report

**Fig. 1:**
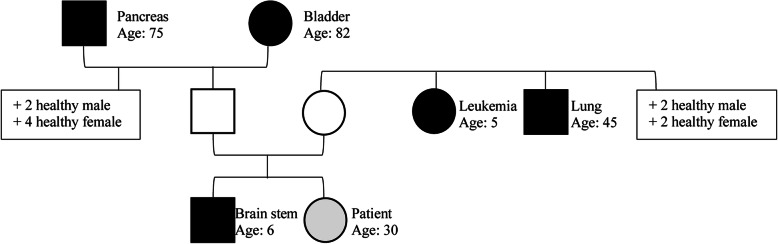
Pedigree with aspect to malignant diseases. Relatives who developed tumor diseases are marked black. Circle, female; square, male

A 30-year-old female (Table 1) was referred to our surgical department because of a giant retroperitoneal and mediastinal mass detected in an ultrasound performed outside of our facility as well as in CT imaging. Her medical history prior to admission was unremarkable, including no long-term medications and no reported pre-existing medical conditions. The initial symptoms were transient nausea, diarrhea, and crampy, left lower quadrant abdominal pain persisting for 2 weeks with a rough palpable mass in physical examination. Family history was positive for cancer, including 5 first- and second-degree relatives (Fig. 1).

**Table 1:** Table of patient characteristics

Age	30
Sex	Female
BMI	31.60
Height (m)	1.64
Weight (kg)	85
CCI	0
ECOG	0
Smoking	None
Drugs	None

*BMI* Body mass index, *CCI* Charlson Comorbidity Index, *ECOG* Eastern Cooperative Oncology Group Performance Index

**Fig. 2:**
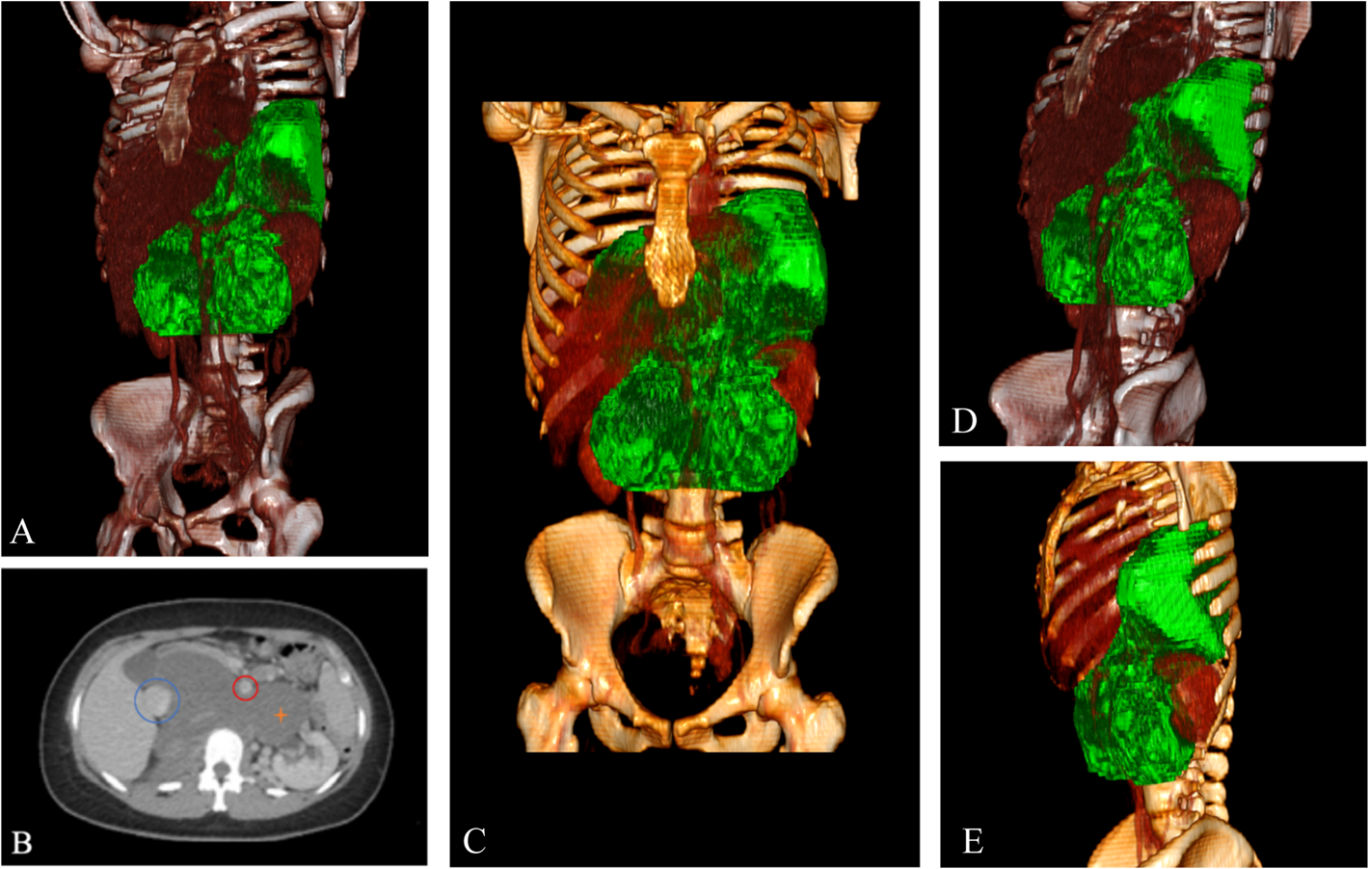
CT images and 3D reconstruction. The GN (green) extends from the retroperitoneal space through the aortic hiatus into the posterior mediastinum and surrounds (**a**, **c**–**e**) and displaces (**b**) Inferior vena cava , aorta , tumor

Initial laboratory examination revealed inflammatory and tumor markers within normal limits. Further CT imaging demonstrated the involvement of the abdominal and thoracic cavities with tumor mass expansion from the retroperitoneum through the aortic hiatus to the posterior mediastinum with a concordant displacement of the major central vessels (Fig. 2).

Presurgical CT-guided biopsy verified a benign ganglioneuroma. Thus, complete surgical resection was recommended in a symptomatic patient. Total resection (TR) of a 35 × 25 × 25 cm, 2550-g tumor via laparotomy and partial diaphragm incision were performed successfully.

**Fig. 3:**
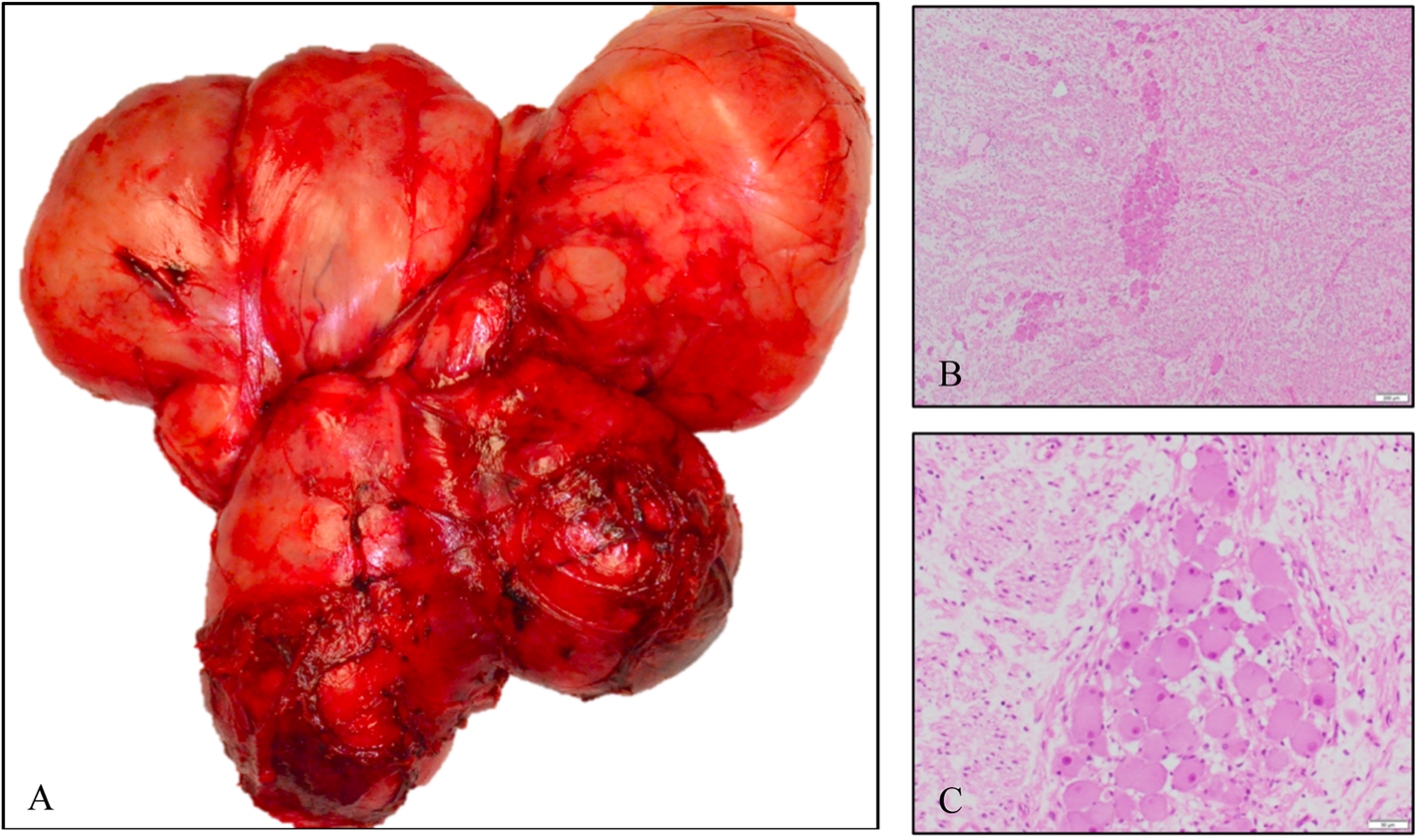
Macroscopic and microscopic findings. **a** Macroscopic specimen of the in toto resected GN. **b**, **c** H&E-stained histopathological images of the central tumor portion with ganglion cells and stromal tissue as well as myxofibrotic and fat tissue in the transition zone. H&E, hematoxylin and eosin staining

Postoperative histological analysis confirmed the diagnosis (Fig. 3). Surgically challenging aspects resulted from the bilateral tumor invasion of the retroperitoneum and the mediastinum through the aortic hiatus, as well as the tumor-related displacement of the abdominal aorta, the mesenteric vessels, and the inferior vena cava. Additionally, the tumor grew between the inferior vena cava and aorta, with the need of comprehensive separation up to the aortic hiatus, where the tumor entered the posterior mediastinum on both sides. Despite the necessity of partial resection of several lumbar aortic vessels due to perivascular tumor growth, postoperative functioning of the patient showed no signs of neurologic impairment. Regular wound drainages (two located intraabdominally and one in the thoracic space) could be removed at day 3 with an output of less than 100 ml. Unfortunately, the patient developed a chylous ascites which required continuous fluid drainage via a PleurX™ system, which was placed retroperitoneally on day 9 postoperatively. This was not completely unexpected due to the vast extent of lymph vessel resection. Supportive medium-chain triglycerides (MCT) diet improved the drain output, such that the PleurX™ drain could be removed at day 27 postoperatively, and the patient was discharged on day 32 postoperatively. Afterwards, no further medication or therapy was needed. At the 6-month follow-up, the patient reported no negative impacts and no restrictions on her activities of daily living in comparison with her preoperative status.

### Systematic literature review

**Fig. 4:**
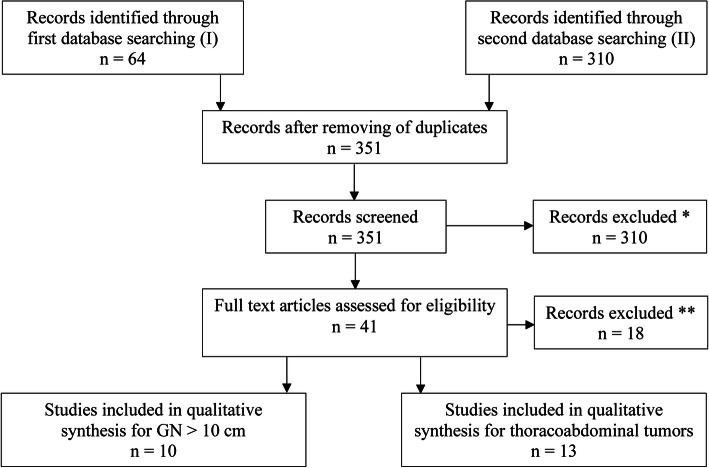
PRISMA consort diagram of how the literature was screened. *Trivial cases of gastric or esophageal cancers as well as spinal cord surgery, which are naturally located thoracoabdominal (e.g., T1–L2 in spinal dumbbell tumors), were excluded. **Mostly thoracoabdominal approach for solely intraabdominally located tumors

A total of 64 papers fulfilled the MESH terms for big resected ganglioneuroma from June 1957 to January 2020. Subsequently, 13 of those reported cases were thoracoabdominal tumors and 10 were GN over 10 cm (Table 2, Fig. 4).

**Table 2:** Systematic literature review for large resected GN (> 10 cm max. diameter)

Year^a^	Age	Sex	Localization	DM	Procedure (^c^)	Complications	Relapse (FU)
2018 [6]	42	♀	Thoracic	23	Open TR	None	n.a.
2006 [25]^b^	35	♂	Retroperitoneal	22	Open TR	None	No (48)
2017 [3]	21	♂	Retroperitoneal	21.5	Open TR	None	No (12)
2013 [26]	18	♂	Retropharyngeal	19	Open TR	None	No (7)
2011 [27]	53	♂	Retroperitoneal	19	Open TR	None	No (24)
2019 [1]	4	♂	Retroperitoneal	17.3	Open PR	None	n.a.
2017 [28]	5	♂	Mediastinal	16	Open TR	None	n.a.
2016 [29]	42	♀	Retroperitoneal	14.5	Open TR	None	No (12)
2007 [30]	23	♀	Retroperitoneal	13	Lap. TR	None	No (12)
2013 [31]	12	♀	Presacral	12	Open TR	None	No (12)
2017 [32]	12	♀	Thoracic	12	Open PR (2)	None	No (12)
2014 [33]	66	♀	Thoracic	12	n.a.	Pain, hypertension	No (36)
2016 [5]	12	♀	Retroperitoneal	13	Open PR (8)	None	n.a.

*FU* Follow-up in months, *TR* Total resection, *PR* Partial resection, *DM* Maximum diameter (in cm)

^a^Citations in brackets

^b^Article in Japanese

^c^Completion in weeks

None of these large (> 10 cm) resected GN showed expansion to both body cavities. Therefore, the natural cap of tumor mass growth was assumed to be limited by the provided space before any symptom onset was noticed by the patient. The largest GN currently reported was 23 cm in diameter [6]. Thus, our case currently ranks as the largest resected ganglioneuroma. Regarding other aspects of the literature, the gender balance was almost even in the reported cases with 7:6 in favor of women. Tumors were located as follows: 7 retroperitoneal, 4 thoracic, and one retropharyngeal as well as one presacral case. A laparoscopic approach was only conducted once concordant with an adrenalectomy, whereas open surgical procedures were performed in the remaining 12 cases. Total resection, on the other hand, was performed in 10 out of 13 cases. In the remaining three cases, one was removed piecemeal in one procedure, whereas a second surgical approach was necessary in the remaining two cases [5, 32]. Twelve out of 13 papers reported the absence of surgical complications or postoperative unexpected events. Unexpected postoperative pain and hypertensive disorders were observed in one patient. Moreover, no relapse was seen at a mean follow-up period of 19.44 (min. 7, max. 48, standard deviation 13.88) months.

A second literature review with extension to all resected tumors with thoracoabdominal expansion was conducted to match our case. A total of 96 papers fulfilling the terms <tumor>, <mediastinum>, <retroperitoneum>, and <resection> as well as 214 papers fulfilling the MESH terms <thoracoabdominal>, <tumor>, and <resection> were evaluated. All tumors that are naturally located thoracoabdominal (e.g., esophagus, stomach) as well as spinal cord (e.g., dumbbell) tumors were neglected in this review. The review resulted in a total of 10 papers and 11 cases of tumors with mediastinal and retroperitoneal expansion that are listed in Table 3.

**Table 3:** Systematic literature review for resected thoracoabdominal growing tumors

Year^a^	***n***	Entity	Age (years)	Sex	Symptoms	DM	Procedure	Complications	Relapse (FU)
2011 [34]	1	Liposarcoma	39	♂	Chest pain	40	Open TR	None	None (14)
2019 [35]	1	Schwannoma	58	♂	Cough	20.2	Open TR	Partial lung expansion	None (n.a.)
2019 [36]	1	GN	10	♀	Cough	8	Lap. TR	None	None (12)
2019 [37]	1	Neuroblastoma	24	♂	Chest pain	7	Open TR	None	None (12)
2019 [38]	1	Sarcoma	74	♀	None	3	Open TR	None	None (2)
2014 [39]	1	GNB	17	♂	n.a.	n.a.^b^	Open TR	n.a.	n.a.
2010 [40]	1	Teratoma	1	♂	n.a.	n.a.^b^	Open TR	None	None (84)
2009 [41]	1	Osteochondroma	17	♂	n.a.	n.a.^b^	Open TR	n.a.	n.a.
2004 [42]	1	n.a.	n.a.	n.a.	n.a.	n.a.^b^	n.a.	n.a.	n.a.
1993 [43]	2	Neuroblastoma	n.a.	n.a.	n.a.	n.a.^b^	n.a.	n.a.	n.a.

*n* case number, *TR* total resection, *PR* partial resection, *DM* maximum diameter (in cm), *FU* follow-up in months

^a^Citation in brackets

^b^Exact max. size unknown, but < 10 cm according to the provided imaging

According to our research, the largest resected tumor ever appears to be a liposarcoma in a 39-year-old man presenting with chest pain in 2011 with a size of 40 cm [34]. Open total resection was performed safely without peri- or postoperative complications. Thus, our case currently ranks as the second largest thoracoabdominal tumor. Gender relation within this research was 6:2 in favor of men, whereas the mean age was 30 years, but again appeared very heterogeneously distributed (min. 1, max. 74, standard deviation 25.12). Laparoscopic approach was only performed once in a case of a small ganglioneuroma (8 cm), with the remaining procedures being performed open. Nevertheless, total resection was feasible in all reported cases accessible.

Surgical complications were only reported in one case report of a schwannoma mainly located at the posterior mediastinum. Furthermore, not a single case of relapse was seen at a mean follow-up period of 24.8 (min. 2, max. 84, standard deviation 33.43) months.

## Discussion

This study presents novel findings in three aspects. First, we report the resection of the largest ganglioneuroma to date (23 vs. 35 cm). GN growth is usually limited by the provided space in the body cavity and the resulting symptoms. Tumors exceeding 10 cm in diameter are very rare in a disease that occurs mostly in children. Of particular interest is the expansion of the tumor through the aortic hiatus into the thoracic cavity without exerting pressure to the surrounding organs. This is an uncommon finding in GN.

Literature research resulted in 13 cases of resected GN of more than 10 cm, with the largest tumors in adults of which the majority were located in the abdomen.

Second, we report the second largest tumor resected with expansion into both the abdominal and thoracic cavities with the limitation of excluding tumors with per se thoracoabdominal growth like dumbbell tumor of the thoracolumbar spine or esophagus or gastric carcinoma. The literature review resulted in only 2 cases of thoracoabdominal GN. The first was reported in a 3.5-year-old girl in 2003, where surgery was skipped due to the highly challenging anatomy and the parents’ will [44]. The second case was reported quite recently in November 2019 in a 10-year-old girl with a paraspinal GN extending from T9–L1, and thus fulfilling the definition of a thoracoabdominal mass. It was also reported to be the first resection of a thoracoabdominal ganglioneuroma [45]. This case showed no involvement of the major vessels, but the described tumor was located close to the right renal artery. The mass showed an expansion of about 8 cm, and TR was obtained solely through the laparoscopic abdominal approach with a small incision and afterwards suture of the diaphragm. Therefore, thoracic involvement was minimal. The current largest thoracoabdominal tumor, a 40-cm liposarcoma, was resected in 2011 via laparotomy and thoracotomy [34]. The undiscussed cases were much smaller in size (Table 3). Additionally, most of these rare cases are presented in case reports without systematic structures such as CARE guidelines and therefore lack quality criteria for a comprehensive comparison. At the time of writing, there does not exist a single case series of big resected ganglioneuromas or tumors with characteristics discussed in this report. Thus, lacking systematic procedure evaluation, the surgical approach depends on the surgeon’s choice as evidence is limited.

Third, removal of big thoracoabdominal tumors may be challenging. A laparoscopic approach may be considered but is restricted to selected smaller tumors. Moreover, the required incision for the final in toto tumor removal from the abdominal cavity would render prior minimally invasive efforts futile. Preoperative diagnostic imaging and functional tests are mandatory to evaluate, for example, tumor vessel invasion like in our case [1], spinal cord invasion [18–21, 32], or potential hormone-producing tumors especially in retroperitoneal/adrenal cases [2, 13–17]. Approaches differ markedly depending on the center’s expertise. This is not surprising as the literature merely consists of case reports. Only one out of 13 cases for resection of big GN and one out of 10 cases for thoracoabdominal tumors presented with peri- or postoperative complications, respectively. We assume that perioperative complications are underreported with respect to the extended resections necessary for tumor removal. In our case, a common complication of extended thoracoabdominal surgical procedures, a chyle leak, occurred. Avoidance of re-operation was achieved with a temporary continuous wound fluid drainage and supportive medium-chain triglycerides (MCT) diet.

In summary, early total resection for symptom control and prevention of potential malignant degeneration as well as regular imaging control during follow-up is recommended, although relapse is rare.

### Patient perspective

“Thinking of the big scar remaining from laparotomy frightened me in a stigmatizing way. Additionally, recovery from surgery took some time and the need of prolongated wound fluid drainage made me feel desperate, but in the end, I could return to normal life without any restrictions.”

### Limitations of the study

Although we tried to limit potential reporting and systematic research bias by adhering to the PRISMA and CARE guidelines, we are aware of the possibility of inadvertently omitting papers not meeting our MeSH terms.

To our knowledge, there is no standard definition for large ganglioneuroma. The given cutoff (over 10 cm) represents a subjective threshold we used to provide a complete overview of a manageable and easily understandable cohort (the biggest GN that had been resected in the past).

## Supplementary information

**Additional file 1: Supplementary File 1.** CARE checklist of information to include when writing a case report.

**Additional file 2: Supplementary File 2.** PRISMA checklist of information to include when writing a systematic review.

## Data Availability

The datasets used and/or analyzed during the current study are available from the corresponding author on reasonable request.

## Abbreviations

GN: Ganglioneuroma
CT: Computed tomography
MRI: Magnetic resonance imaging
VIP: Vasoactive intestinal peptide
VMA: Vanillyl mandelic acid
TR: Total resection
PR: Partial resection
H&E: Hematoxylin and eosin
T: Thoracic vertebra
L: Lumbal vertebra
GNB: Ganglioneuroblastoma
MCT: Medium-chain triglycerides
