# Supplementary material for: Total resection of a giant retroperitoneal and mediastinal ganglioneuroma—case report and systematic review of the literature
Source: World J Surg Oncol. 2020 Sep 18;18:248. doi: 10.1186/s12957-020-02016-1 (PMC7501651; doi:10.1186/s12957-020-02016-1)
Supplement: Supplementary file 1 — Additional file 1: Supplementary File 1. CARE checklist of information to include when writing a case report. [file 12957_2020_2016_MOESM1_ESM.pdf]

CARE case report guidelines

CARE Checklist of information to include when writing a case report

(cc) BY-NC-ND

Topic	Item	Checklist item description	Reported on Line
Title	1	The diagnosis or intervention of primary focus followed by the words “case report”	2
Key Words	2	2 to 5 key words that identify diagnoses or interventions in this case report, including "case report"	24
Abstract (no references)	3a	Introduction: What is unique about this case and what does it add to the scientific literature?	55-59
	3b	Main symptoms and/or important clinical findings	45-46
	3c	The main diagnoses, therapeutic interventions, and outcomes	45-50
	3d	Conclusion—What is the main “take-away” lesson(s) from this case?	55-59
Introduction	4	One or two paragraphs summarizing why this case is unique (**may include references**)	98-101
Patient Information	5a	De-identified patient specific information.	Tbl. 1
	5b	Primary concerns and symptoms of the patient.	127-129
	5c	Medical, family, and psycho-social history including relevant genetic information	Tbl. 1+Fig. 1
	5d	Relevant past interventions with outcomes	126-127
Clinical Findings	6	Describe significant physical examination (PE) and important clinical findings.	129
Timeline	7	Historical and current information from this episode of care organized as a timeline	124-129
Diagnostic Assessment	8a	Diagnostic testing (such as PE, laboratory testing, imaging, surveys).	125-134 + 145
	8b	Diagnostic challenges (such as access to testing, financial, or cultural)	---
	8c	Diagnosis (including other diagnoses considered)	145
	8d	Prognosis (such as staging in oncology) where applicable	---
Therapeutic Intervention	9a	Types of therapeutic intervention (such as pharmacologic, surgical, preventive, self-care)	146-147
	9b	Administration of therapeutic intervention (such as dosage, strength, duration)	148-154
	9c	Changes in therapeutic intervention (with rationale)	156-162
Follow-up and Outcomes	10a	Clinician and patient-assessed outcomes (if available)	163-164
	10b	Important follow-up diagnostic and other test results	163-164
	10c	Intervention adherence and tolerability (How was this assessed?)	---
	10d	Adverse and unanticipated events	151-154, 157
Discussion	11a	A scientific discussion of the strengths AND limitations associated with this case report	273-279
	11b	Discussion of the relevant medical literature **with references**.	171-218
	11c	The scientific rationale for any conclusions (including assessment of possible causes)	243-261
	11d	The primary “take-away” lessons of this case report (without references) in a one paragraph conclusion	55-59
Patient Perspective	12	The patient should share their perspective in one to two paragraphs on the treatment(s) they received	267-270
Informed Consent	13	Did the patient give informed consent? Please provide if requested	Yes [X] No [ ]
